# Impact of liposomal bupivacaine on subjective recovery quality after surgery: a meta-analysis of randomized controlled trials

**DOI:** 10.3389/fmed.2025.1655756

**Published:** 2025-10-08

**Authors:** Longyi Zhang, Shun Yang, Xianchun Liu, Linlin Chen, Xuelei Zhou, Wei Mao, Li Zhao, Linji Li, Ying Xie

**Affiliations:** Department of Anesthesiology, The Second Clinical Medical College, North Sichuan Medical College, Beijing Anzhen Nanchong Hospital of Capital Medical University & Nanchong Central Hospital, Nanchong, China

**Keywords:** liposomal bupivacaine, quality of recovery, pain, postoperative nausea and vomiting, surgery

## Abstract

**Background:**

The quality of recovery (QoR) is a multidimensional concept used to evaluate the restoration of physical and psychological functions after surgery. It is a key measure for assessing surgical efficacy, anesthetic modalities, and the effectiveness of perioperative interventions. Liposomal bupivacaine (LB), a long-acting local anesthetic recently introduced, is characterized by its sustained-release profile and prolonged analgesic effect. However, empirical findings regarding the impact of LB on the quality of postoperative recovery remain inconclusive.

**Methods:**

On 5 March 2025, we conducted a systematic search of the PubMed, Embase, Web of Science, Cochrane Library, Scopus, CNKI, and VIP databases to identify randomized controlled trials (RCTs) assessing the efficacy of LB in postoperative recovery. Eligible studies compared postoperative recovery outcomes between patients receiving LB and those in control groups who did not receive LB. The primary outcome of interest was the QoR score measured 72 h after surgery. Secondary outcomes included QoR scores at 24 and 48 h after surgery; pain scores at rest at 24, 48, and 72 h after surgery; incidence of postoperative nausea and vomiting (PONV); cumulative morphine-equivalent opioid consumption within 72 h after surgery; and patient satisfaction.

**Results:**

This meta-analysis included 11 RCTs comprising a total of 1,357 patients. The findings indicate that the LB group showed a statistically significant improvement in overall QoR scores 72 h after surgery [standardized mean difference (SMD): 0.52; 95% confidence interval (CI): 0.20, 0.85; *P* = 0.00]. Furthermore, LB use was associated with significantly reduced pain scores at 24, 48, and 72 h after surgery. The LB group also exhibited lower cumulative morphine-equivalent opioid consumption within 72 h after surgery, a reduced incidence of PONV, and significantly higher patient satisfaction scores.

**Conclusion:**

The use of LB was associated with improved QoR scores within 72 h after surgery, a reduced incidence of PONV, and decreased opioid consumption. These findings suggest that LB not only improves overall postoperative recovery but also mitigates associated adverse effects, thereby contributing to improved patient satisfaction and a more streamlined recovery trajectory. Nevertheless, further research is needed to assess its long-term efficacy and broader clinical applicability.

**Systematic review registration:**

[https://www.crd.york.ac.uk/prospero/], identifier [CRD420251003585].

## 1 Introduction

Postoperative physiological and psychological changes, driven by factors such as tissue trauma, inflammatory responses, and pain, frequently disrupt the recovery process. These disturbances not only increase patient discomfort, but also prolong hospital stays, and exacerbate the economic burden (1). In this context, the quality of recovery (QoR) has gained recognition as a multidimensional, patient-centered outcome that evaluates key domains including pain relief, physical function, emotional wellbeing, and overall comfort (2, 3). Validated instruments like the QoR-15 and QoR-40 are widely used to assess perioperative interventions across diverse surgical settings (4–6). In the context of the expanding adoption of Enhanced Recovery After Surgery (ERAS) protocols, optimizing postoperative analgesia, minimizing complications, and facilitating accelerated recovery are central goals of modern perioperative care (7–9).

Although conventional local anesthetics such as bupivacaine and ropivacaine are widely employed for postoperative analgesia, their relatively short duration of action is often insufficient to manage pain during the critical early phase of postoperative recovery (10–12). To extend analgesic efficacy, opioids are frequently used as adjuncts; however, their use significantly increases the risk of postoperative nausea and vomiting (PONV) and other analgesia-related adverse effects, thereby undermining the goals of ERAS protocols. Liposomal bupivacaine (LB) is a novel long-acting local anesthetic developed with a multilamellar liposomal delivery system that allows sustained release and provides analgesia for up to 72 h (13, 14). It may reduce opioid consumption, lower the incidence of PONV, and improve QoR (15). Several previous meta-analyses have evaluated LB’s effects on postoperative pain and opioid consumption, showing modest benefits but with inconsistent conclusions (16–18). However, no prior meta-analysis has systematically examined LB’s impact on overall postoperative QoR. To address this gap, we conducted a meta-analysis to evaluate the effects of LB on postoperative QoR, analgesic efficacy, and PONV, aiming to provide more comprehensive evidence to guide perioperative pain management within ERAS frameworks.

## 2 Methods

This systematic review is registered with the International Prospective Register of Systematic Reviews (PROSPERO) under registration number CRD420251003585. The study strictly adheres to the guidelines outlined in the Preferred Reporting Items for Systematic Reviews and Meta-Analyses (PRISMA) statement (19).

### 2.1 Search strategies

On 5 March 2025, we conducted a systematic search across the following databases: PubMed, Embase, Web of Science, Cochrane Library, Scopus, CNKI, and VIP. The search strategy combined Medical Subject Headings and free-text terms, with keywords including “liposomal bupivacaine,” “Exparel,” “quality of recovery,” and “functional recovery,” among others. To ensure the inclusion of all relevant studies, no restrictions were applied regarding publication date or language. Rigorous inclusion criteria were applied to select randomized controlled trials (RCTs) assessing postoperative recovery quality related to LB. Two independent reviewers reviewed the titles, abstracts, and full texts of the retrieved studies. Discrepancies during the evaluation process were resolved through discussion and consensus. In cases of unresolved discrepancies, a third reviewer makes the final decision.

Inclusion criteria: (1). Studies involving patients who have undergone surgical procedures. (2). RCTs. (3). The intervention group receiving LB and a control group not receiving LB. (4). Use of the QoR scale (QoR-15, QoR-40) as an outcome measure, with reported outcomes using the relevant scoring results.

Exclusion criteria: (1). Non-randomized study designs, including observational and cohort studies. (2). Case reports. (3). Review articles. (4). Protocols of clinical trials. (5). Studies lacking QoR scores or with incomplete reporting of relevant data. (6). Articles with inaccessible full texts, in which attempts to contact the authors were unsuccessful. (7). Dissertations or theses.

### 2.2 Data collection and extraction

Two independent reviewers construct data tables and extracted pertinent data. In case of discrepancies, the third reviewer make the final decision. The extracted data included the following: study characteristics, including the first author, publication year, country or region of the study, type, dose, concentration, and administration route of the local anesthetic used, sample size, and anesthesia techniques employed; demographic characteristics of the participants, including patient age, type of surgery, duration of surgery, and primary and secondary outcome measures, such as QoR-15 or QoR-40 scores at 24, 48, and 72 h postoperatively; pain scores at the corresponding time points in a resting state; incidence of PONV; total morphine consumption in the first three postoperative days (only data reported as “morphine equivalents” were extracted). If the data were presented as medians and interquartile ranges, the method proposed by Wan et al. was used to estimate means and standard deviations (20). These data were primarily obtained from quantitative data in tables and figures. For data presented graphically, we used the WebPlotDigitizer tool (version 4.6; A. Rohatgi, Pacifica, CA, United States) to extract the relevant information.

#### 2.2.1 Bias and quality assessment

In the studies included in this analysis, two reviewers independently assess the risk of bias in the RCTs using the Cochrane Collaboration’s tool for assessing risk of bias in randomized trial 2 (RoB 2) tool (21). Standard risk of bias domains included personnel and outcome assessors, blinding of participants, allocation concealment, random sequence generation, incomplete outcome data, and selective reporting. Any disagreements were resolved through discussions between the two reviewers, and if consensus could not be reached, the third reviewer made the final decision. To evaluate the quality of evidence for each outcome, we applied the Grading of Recommendations, Assessment, Development, and Evaluation (GRADE) methodology (22). Using this methodology, the quality of evidence was classified as very low, low, moderate, or high.

### 2.3 Data analysis methods

Data analysis was performed using Stata 17.0 software. The Random-effects Restricted Maximum Likelihood model was employed for the analysis. For continuous variables, mean difference (MD), standardized mean difference (SMD), and their corresponding 95% confidence intervals (CI) were calculated. When continuous outcomes were assessed using different measurement scales across studies, we used the SMD to allow for appropriate pooling of data. The effect sizes were interpreted as follows: small effect size (SMD: 0.2–0.5), moderate effect size (SMD = 0.5–0.8), and large effect size (SMD ≥ 0.8) (23). For binary variables, log odds ratio [log(OR)] and its 95% CI were computed. Although the log odds ratio [log(OR)] and the odds ratio (OR) reflect the same relationship, the log(OR) is preferred due to its stability and normal distribution characteristics, particularly when dealing with smaller sample sizes (24, 25). A random-effects model was employed in all analyses to account for both within-study and between-study variability, thus providing a more accurate reflection of real-world conditions. Between-study heterogeneity was assessed using τ^2^ (tau-squared) and I^2^ (I-squared) (26). I^2^ values of 0%, 25%, 50%, and 75% were interpreted as indicative of no, low, moderate, and high heterogeneity, respectively. Subgroup analyses were performed based on surgical type, surgical duration (i.e., ≤ 90 min vs. > 90 min), outcome measurement type (i.e., QoR-15 vs. QoR-40), age (i.e., < 60 years vs. > 60 years), LB dose (i.e., 133 vs. 266 mg), study country, whether LB was used alone, and the administration route of LB to investigate the sources of heterogeneity and inform clinical practice. Sensitivity analysis was conducted using the “leave-one-out” method to assess the robustness of the meta-analysis results. Specifically, each study was excluded individually to evaluate its influence on the pooled effect estimate. Additionally, a re-analysis was performed after excluding studies deemed to have a high risk of bias, in order to assess the impact of study quality on the results. The sources of heterogeneity were further explored using Galbraith plots (27). Points outside the 95% confidence interval (CI) on the plot were identified as potential sources of heterogeneity (26). Considering Cochrane’s guidelines (28), as the number of included studies for each outcome was less than 10, we did not assess publication bias either visually (funnel plot) or statistically (Egger’s test).

## 3 Results

### 3.1 Study inclusion

**FIGURE 1:**
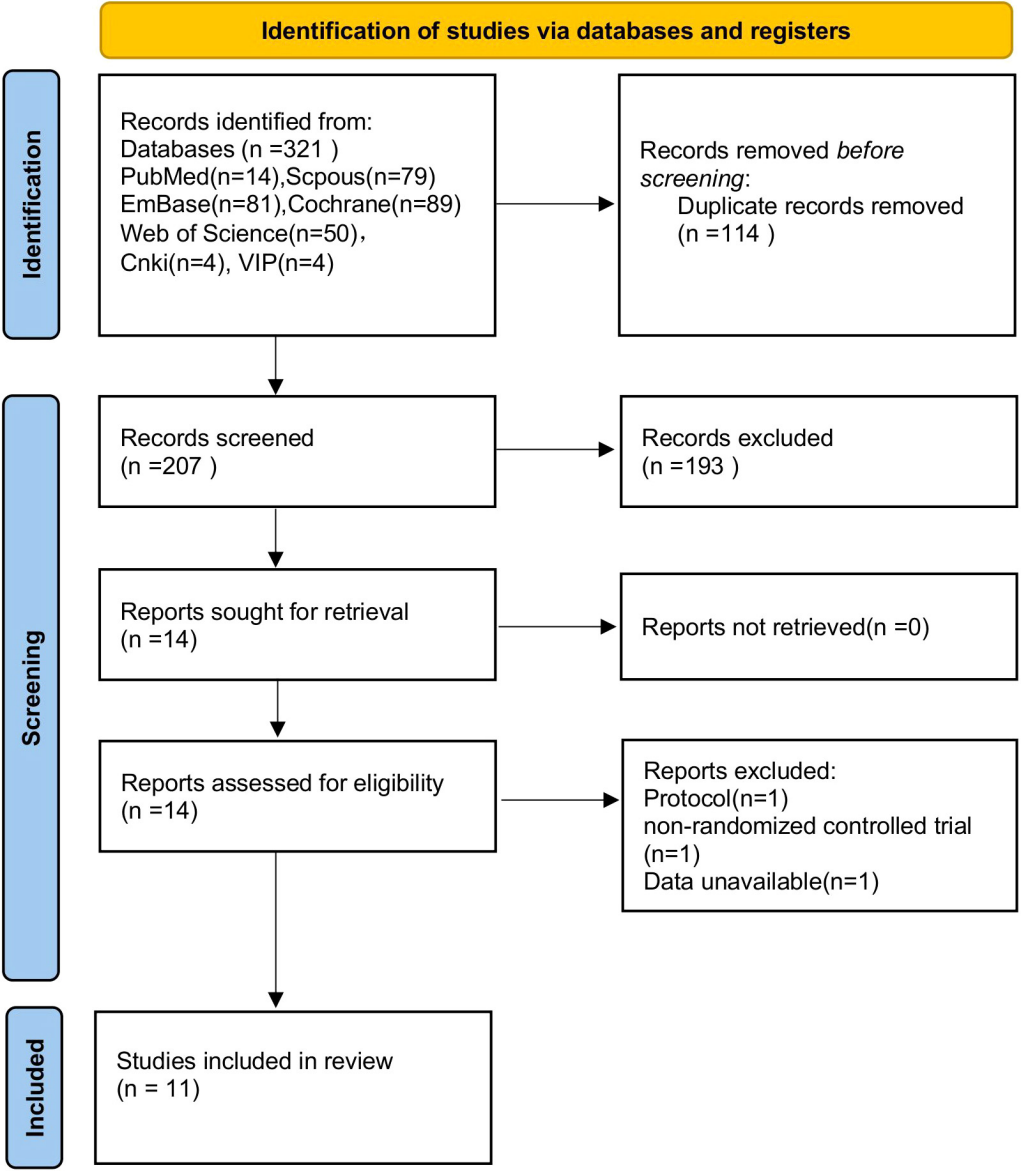
Flow diagram of study selection.

**FIGURE 2:**
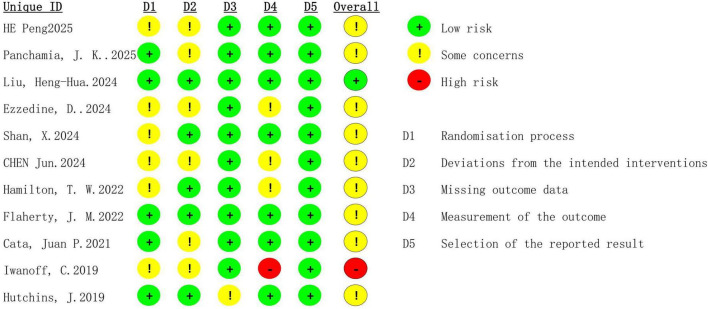
Cochrane Collaboration Risk of Bias 2 summary: evaluation of bias risk items for each included study. Green circle, low risk of bias; red circle, high risk of bias; yellow circle, unclear risk of bias.

A total of 321 articles were initially identified. After removing duplicates, screening titles and abstracts, and conducting full-text reviews, 11 RCTs were included in the final analysis. Details of the selection process are illustrated in the PRISMA flow diagram (Figure 1). The risk of bias of included studies is summarized in Figure 2.

### 3.2 Characteristics of included studies

The detailed characteristics of the included studies are presented in Table 1. This analysis included 11 RCTs. Of these, nine studies employed general anesthesia (29–37), one study employed neuraxial anesthesia (38), and one study did not specify the anesthesia modality (39). In 4 studies, LB was administered as a monotherapy (30–33), while in six studies, LB was combined with other local anesthetics (29, 34–37, 39). One study included two intervention arms—one receiving LB alone and another receiving a combination regimen (38). Regarding dosage, six studies administered 266 mg of LB (30, 32, 33, 35, 36, 39), whereas five studies administered 133 mg (29, 31, 34, 37, 38). Postoperative analgesia strategies varied: seven studies implemented a multimodal analgesia approach (29, 30, 32–35, 38), while four studies adopted a unimodal strategy (31, 36, 37, 39).

**TABLE 1:** Study characteristics.

References	Surgical type	Scale design	Experimental			Control		
			Age (years)/sample size	Surgical time (min)	Block technique	Age (years)/sample size	Surgical time (min)	Block technique
Peng et al. (30)	Radical gastrectomy	QoR-15	69.00 ± 6.94/30	234.73 ± 11.77	100 ml 133 mgLB + 10 ml normal saline anterior quadratus lumborum block at the lateral arcuate ligament on each side	69.13 ± 7.08/30	236.20 ± 15.22	20 ml 0.375% ropivacaine anterior quadratus lumborum block at the lateral arcuate ligament on each side
Panchamia et al. (29)	Shoulder arthroplasty	QoR-15	71 ± 7.6/39	65 ± 14.6	10 ml 133 mgLB + 5 ml 5% bupivacaine interscalene brachial plexus block on each side	70 ± 7.6/44	65 ± 11.9	0.2% bupivacaine at 8 mL/h continuous interscalene infusion
Liu et al. (38) Heng-Hua (Mixture) 2025	Cesarean delivery	QoR-15	32 ± 4.8/49	45 ± 15	10 ml 133 mgLB + 10 ml 0.25% bupivacaine TAP block on each side	31 ± 4.9/25	42 ± 11.8	25 mg bupivacaine TAP block on each side
Liu et al. (38), Heng-Hua (Liposomal) 2025	Cesarean delivery	QoR-15	31 ± 5.5/49	43 ± 9.2	10 ml 133 mgLB + 10 ml normal saline TAP block on each side	31 ± 4.9/24	42 ± 11.8	25 mg bupivacaine TAP block on each side
Ezzedine, et al. (31)	Sacrospinous ligament suspension	QoR-15	65.3 ± 11.3/38	120.77 ± 31.3	10 ml 133 mgLB pudendal nerve block on each side	63.90 ± 8.85/40	124.98 ± 39.16	No block
Xisheng et al. (32)	Thoracoscopic lung resection	QoR-15	55 ± 14/64	93 ± 60.7	20 ml 266 mgLB + 10 ml normal saline erector spinae plane block	55 ± 14/64	93 ± 60.7	100 mg bupivacaine erector spinae plane block
Jun et al. (33)	Laparoscopic gynecological surgery	QoR-40	55.6 ± 15.3/60	Not specified	10 ml 133 mgLB + 10 ml normal saline TAP block on each side	56.48 ± 16.61/60	Not specified	20 ml 0.25% ropivacaine TAP block on each side
Hamilton et al. (39)	Knee replacement	QoR-40	68.9 ± 10.1/267	110 ± 29.1	120 ml 266 mg LB + 100 mg bupivacaine periarticular infiltration	69.0 ± 9.3/266	113.9 ± 32.6	100 mg bupivacaine periarticular infiltration
Flaherty et al. (34)	Rotator cuff repair surgery	QoR-15	59.2 ± 11.0/35	87.5 ± 17.7	10 ml 133 mg LB + 10 ml 0.5% bupivacaine single-injection interscalene brachial plexus block	57.6 ± 8.8/35	90.8 ± 20.6	100 mg bupivacaine single-injection interscalene brachial plexus block
Cata et al. (35)	Cytoreductive surgery with hyperthermic intraperitoneal chemotherapy surgery	QoR-15	53 ± 11.2/35	Not specified	266 mg LB + 150 mg bupivacaine 4Q-TAP block	51 ± 41.8/33	Not specified	Bupivacaine 0.075% ± hydromorphone 2–5 mcg/mL or bupivacaine 0.075% ± fentanyl 5 mcg/mL, basal rate 8 mL/h, bolus 3 mL every 10 min TEA
Iwanoff et al. (36)	Midurethral Sling Placement	QoR-15	53.3 ± 10.8/24	73 ± 37.8	266 mg LB + 20 mL of the anesthetic retropubic and suprapubic	51.2 ± 7.6/33	32 ± 27.9	150 mg bupivacaine + 500 mg lidocaine + 20 mL of the anesthetic retropubic and suprapubic
Hutchins et al. (37)	Robotic-assisted and laparoscopic hysterectomy	QoR-15	56 ± 35/31	Not specified	133 mgLB + 25mg bupivacaine + 0.05mg adrenaline TAP block + sham wound infiltration on each side	61 ± 43.5/31	Not specified	25 mg bupivacaine + 0.05 mg adrenaline sham TAP block ± wound infiltration

TAP, transversus abdominis plane; TEA, thoracic epidural analgesia; QoR, quality of recovery; LB, liposomal bupivacaine.

### 3.3 QoR at 72 h

**FIGURE 3:**
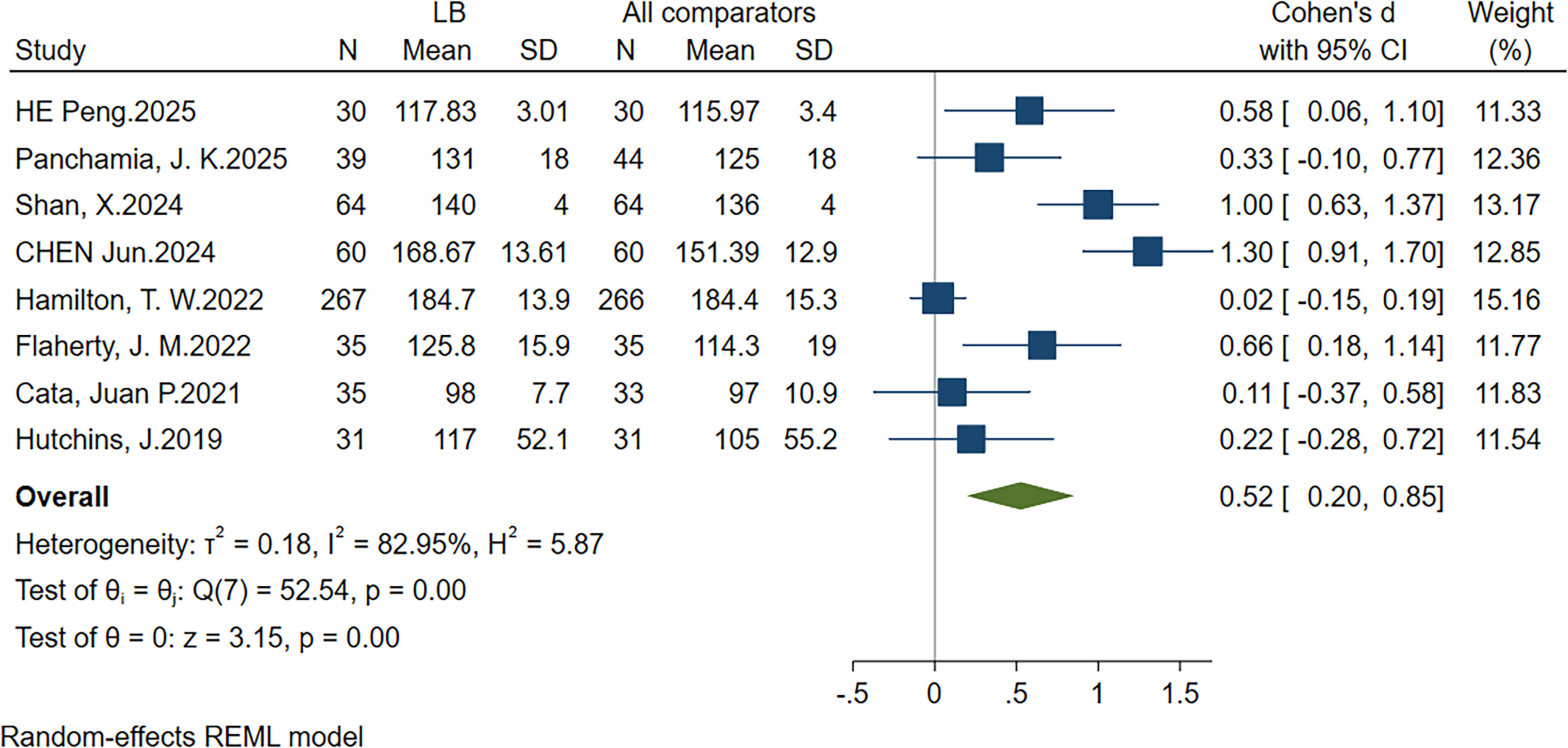
Forest plot showing quality of recovery (QoR) within 72 h after surgery, SD, standard deviation; CI, confidence interval.

Eight RCTs (29, 30, 32–35, 37, 39) reported QoR scores at 72 h postoperatively. The pooled results demonstrated that the LB group exhibited significantly higher QoR scores than the control group (SMD: 0.52; 95% CI: 0.20, 0.85; *P* = 0.00; I^2^ = 82.95%), indicating a statistically significant improvement (Figure 3).

### 3.4 QoR at 24 and 48 h

Nine RCTs (29–33, 35, 36, 38, 39) and five RCTs (29, 33, 35, 38, 39) reported QoR scores at 24 and 48 h postoperatively, respectively. The results indicated no statistically significant differences between the LB and control groups at either time point. At 24 h postoperatively, the QoR scores showed no statistically significant difference (SMD: 0.52; 95% CI: −0.06 to 1.09; *P* = 0.08; I^2^ = 95.18%) (Supplementary Figure 1). Similarly, at 48 h, the difference in QoR scores was also not statistically significant (SMD: 0.22; 95% CI: −0.19 to 0.64; *P* = 0.30; I^2^ = 86.61%) (Supplementary Figure 2).

### 3.5 Resting pain scores

A total of eight studies assessed postoperative resting pain scores. Among them, two studies reported pain using the Visual Numeric Scale (VNS) (31, 35), two used the Numeric Rating Scale (NRS) (29, 33), and four adopted the Visual Analog Scale (VAS) (30, 36, 38, 39).

Meta-analysis results indicated that patients in the LB group reported significantly lower resting pain scores compared to the control group at 24 h postoperatively (*n* = 8 RCTs, SMD = −0.49, 95% CI: −0.87, −0.11, *P* = 0.01, I^2^ = 87.75%) (Supplementary Figure 3), at 48 h (*n* = 8 RCTs, SMD = −0.52, 95% CI: −0.83, −0.22, *P* = 0.00, I^2^ = 80.42%) (Supplementary Figure 4), and at 72 h (*n* = 7 RCTs, SMD = −0.47, 95% CI: −0.79, −0.14, *P* = 0.01, I^2^ = 81.34%) (Supplementary Figure 5).

### 3.6 Cumulative morphine consumption over 3 days postoperatively

Five studies reported the cumulative morphine consumption over the first three postoperative days (29, 32, 34, 37, 39). Compared to the control group, no significant difference was observed in the LB group regarding total morphine consumption over the three days (MD: −3.84 mg; 95% CI: −11.74, 4.06; *P* = 0.34; I^2^ = 62.75%) (Supplementary Figure 6). To further explore the source of heterogeneity, we employed a Galbraith plot (Supplementary Figure 7). After excluding the study by Panchamia et al. (29), and reanalyzing the remaining four studies, we observed that the LB group showed a significant reduction in postoperative morphine consumption compared to the control group (MD: −7.77 mg; 95% CI: −10.09, −5.44; *P* = 0.00; I^2^ = 0%) (Supplementary Figure 8).

### 3.7 PONV incidence

Six studies reported the incidence of PONV (30, 32–34, 37, 38). Compared to the control group, the LB group demonstrated a significant reduction in the incidence of PONV [log(OR): −0.72; 95% CI: −1.22, −0.23; *P* = 0.00; I^2^ = 0%) (Supplementary Figure 9).

### 3.8 Patient satisfaction

Two studies reported patient satisfaction using a numerical rating scale (32, 38). Compared to the control group, the LB group demonstrated a significant increase in patient satisfaction (MD: 1.03; 95% CI: 0.86, 1.21; *P* = 0.00; I^2^ = 0%) (Supplementary Figure 10).

### 3.9 Subgroup and sensitivity analysis

As shown in Table 2, geographical subgroup analysis revealed that studies conducted in China demonstrated a significantly larger effect size (SMD = 0.99; *P* < 0.001), while those from the United States showed a moderate effect (SMD = 0.33; *P* = 0.006). Studies from the United Kingdom exhibited a small and statistically non-significant effect (SMD = 0.02; *P* = 0.813). The differences between these subgroups were statistically significant (*P* = 0.00).

**TABLE 2:** Subgroup analyses of quality of recovery (QoR) at 72 h.

Category	Number of trials	Effect size SMD (95% CI)	I^2^ (%)	*P*-value	Subgroup difference
**Country**					0.00
China	3	0.99 (0.61,1.37)	59.51%	0.000	–
America	4	0.33 (0.10,0.57)	0	0.006	–
Britain	1	0.02 (−0.15,0.19)	NA	0.813	–
**Assessment scale**					0.82
QoR-15	6	0.50 (0.22,0.78)	57.8%	0.001	–
QoR-40	2	0.65 (−0.61,1.91)	97.1%	0.311	–
**Age**					0.13
Average age ≤ 60 years old	5	0.68(0.23,1.12)	61.9%	0.003	–
Average age > 60 years old	3	0.24 (−0.09,0.57)	80.7%	0.159	–
**Surgery time**					0.92
≤ 90 min	2	0.48 (0.16,0.80)	0	0.004	–
> 90 min	3	0.51 (−0.08,1.10)	89.15%	0.135	–
**Types of local anesthetics**					0.00
LB	3	0.99 (0.61,1.37)	59.51%	0.000	–
LB combined conventional local anesthetics	5	0.22 (−0.01,0.45)	44.67%	0.066	–
**Dose**					0.51
133 mg	3	0.40 (0.13,0.67)	0	0.004	–
266 mg	5	0.60 (0.10,1.10)	90.04%	0.036	–
**Administration method**					0.00
Nerve block	6	0.68 (0.32,1.04)	74.72%	0.000	–
Local infiltration	2	0.04 (−0.12,0.20)	0	0.612	–

LB, liposomal bupivacaine.

Regarding the type of assessment scale used, studies utilizing the QoR-15 scale reported a moderate effect (SMD = 0.50; *P* = 0.001), whereas those employing the QoR-40 scale demonstrated a larger but statistically non-significant effect (SMD = 0.65; *P* = 0.311). No significant differences were observed between these subgroups.

Based on the type of surgery revealed that thoracic surgery showed the largest effect (SMD = 1.00; *P* < 0.001), followed by abdominal surgery with a moderate and significant effect (SMD = 0.57; *P* = 0.047). In contrast, orthopedic surgery demonstrated a small and statistically non-significant effect (SMD = 0.28; *P* = 0.145). The differences between these subgroups were statistically significant (*P* = 0.03).

Age-based analysis indicated that participants aged ≤ 60 years exhibited a significantly higher effect size (SMD = 0.68; *P* = 0.003), while those older than 60 years showed a smaller, non-significant effect (SMD = 0.24; *P* = 0.159). However, the difference between age subgroups was not statistically significant.

In terms of surgical duration, procedures lasting ≤ 90 min were associated with a significant effect (SMD = 0.48; *P* = 0.004), whereas those exceeding 90 min showed a non-significant effect (SMD = 0.51; *P* = 0.135). Subgroup differences were not statistically significant.

With respect to intervention type, studies using LB as monotherapy reported a significantly larger effect (SMD = 0.99; *P* = 0.000) compared to those combining LB with traditional local anesthetics (SMD = 0.22; *P* = 0.066). The difference between these subgroups was statistically significant (*P* = 0.00).

In the dosage subgroup analysis, a 266 mg dose of LB was associated with a larger and more consistent effect than the 133 mg dose (SMD = 0.60; *P* = 0.036), although the intergroup difference did not reach statistical significance.

**FIGURE 4:**
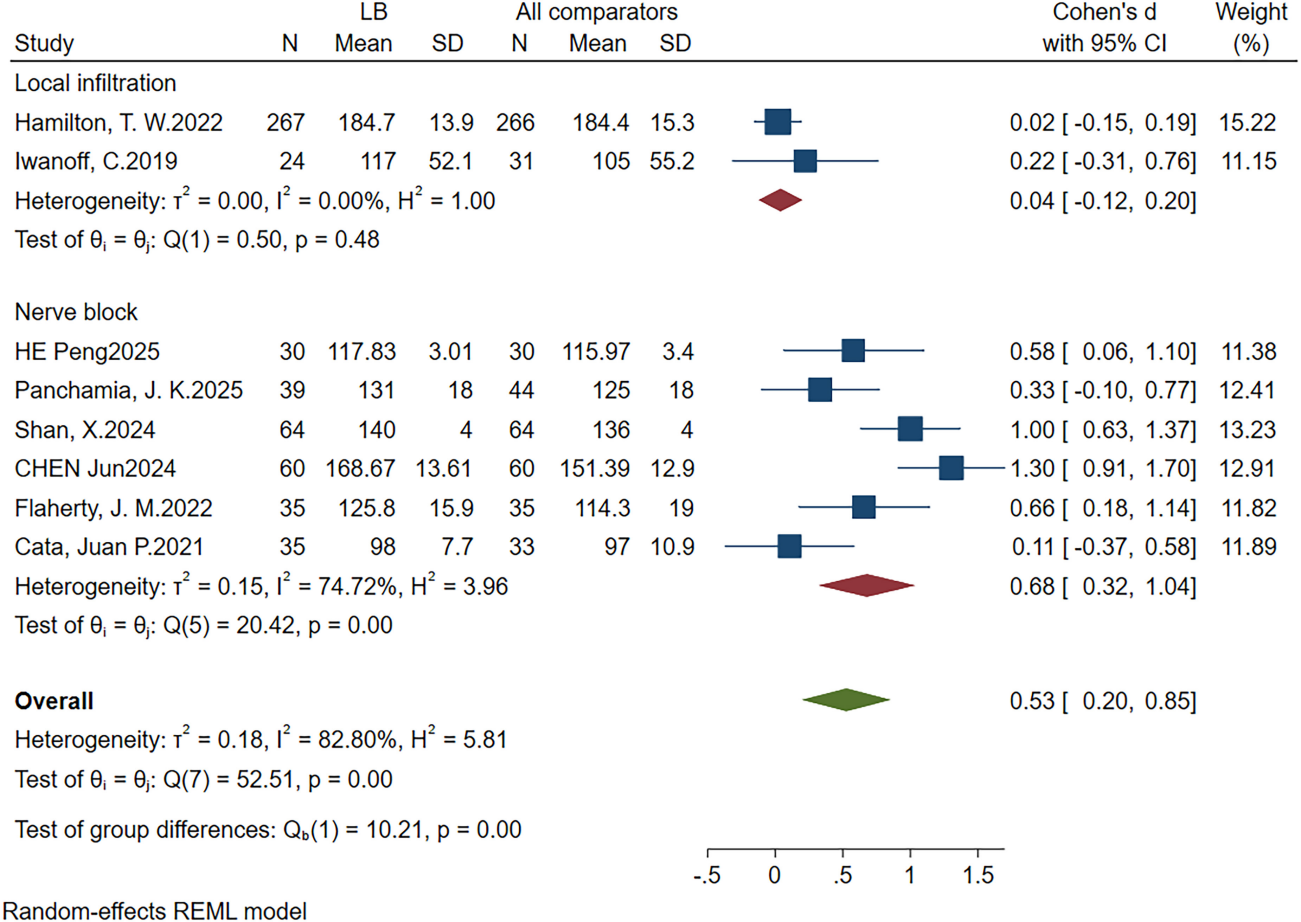
Subgroup analysis forest plot of 72 h postoperative quality of recovery (QoR) score administration mode.

Finally, with respect to the administration route, nerve block techniques demonstrated a statistically significant moderate effect (SMD = 0.68; *P* = 0.000), whereas local infiltration yielded no significant effect (SMD = 0.04; *P* = 0.612). The difference between these subgroups was statistically significant (*P* = 0.00) (Figure 4).

Sensitivity analysis using the leave-one-out method (Supplementary Figures 11–19) demonstrated that most outcome measures remained robust after sequential exclusion of individual studies. Specifically, postoperative QoR scores at 24 and 72 h, resting pain scores, PONV incidence, and patient satisfaction showed no significant changes upon exclusion of any single study However, sensitivity analysis revealed that the exclusion of Cata et al. (35) changed the 48-h QoR score outcome from non-significant to statistically significant (*P*-value shifted from 0.30 to 0.039). Likewise, for cumulative morphine consumption, omitting the study by Panchamia et al. (29) altered the result from non-significant (*P* = 0.34) to highly significant (*P* = 0.000).

### 3.10 Quality assessment

The quality of evidence for the incidence of PONV was rated as high. Evidence supporting postoperative QoR at 24 and 72 h, pain scores, and cumulative morphine consumption within the first 3 days was assessed as moderate. In contrast, the quality of evidence for postoperative QoR at 48 h and patient satisfaction was deemed low (Table 3).

**TABLE 3:** GRADE evidence summary table.

Outcome	Studies included	Participants (LB/control)	Quality of evidence (GRADE)	Importance	I^2^
Postoperative 24-h QoR score	9	655/619	⊕⊕⊕⊝	Important	95.18%
Postoperative 48-h QoR Score	5	464/419	⊕⊕⊝⊝	Important	86.61%
Postoperative 72-h QoR score	8	561/563	⊕⊕⊕⊝	Critical	82.95%
Postoperative 24-h pian score	8	591/555	⊕⊕⊕⊝	Important	87.75%
Postoperative 48-h pian score	8	591/555	⊕⊕⊕⊝	Important	80.42%
Postoperative 72-h pian score	7	556/522	⊕⊕⊕⊝	Important	81.34%
PONV rate	6	33/318 (10.3%)/52/269 (19.3%)	⊕⊕⊕⊕	Critical	0
Postoperative cumulative morphine consumption 3 days	4	397/396	⊕⊕⊕⊝	Important	0
Patient satisfaction	2	162/113	⊕⊕⊝⊝	Important	0

⊕⊕⊕⊕ high quality evidence; ⊕⊕⊕⊝, moderate quality evidence; ⊕⊕⊝⊝, low quality evidence, ⊕⊝⊝⊝, very low-quality evidence. GRADE, Grades of Recommendation, Assessment, Development, and Evaluation; PONV: postoperative nausea and vomiting.

## 4 Discussion

This meta-analysis demonstrated that LB significantly improved QoR scores at 72 h postoperatively, enhanced patient satisfaction, reduced resting pain scores, and lowered the incidence of PONV. Furthermore, LB may contribute to decreased cumulative morphine consumption within the first three postoperative days. However, no statistically significant differences were observed in QoR scores at 24 and 48 h postoperatively. These findings suggest that LB provides multidimensional clinical benefits during early postoperative recovery, particularly by optimizing analgesia, reducing opioid dependence, and enhancing the overall recovery experience.

To the best of our knowledge, this is the first meta-analysis to specifically evaluate the impact of LB on postoperative QoR. Our findings indicate that LB significantly enhances QoR scores at 72 h postoperatively. Importantly, when stratified by measurement scale, the mean difference in QoR-40 was 8.66—exceeding the established minimal clinically important difference (MCID) of 6.3—suggesting a meaningful benefit in recovery (40). In contrast, while the improvement in QoR-15 (MD = 3.32) reached statistical significance, it did not meet the MCID threshold of 6, indicating limited clinical relevance (41). This highlights the importance of interpreting statistical findings in the context of patient-centered thresholds.

Several mechanisms may account for this effect. First, LB employs a multivesicular liposomal delivery system, enabling sustained and gradual drug release at the site of administration, with analgesic effects extending up to 72 h. This prolonged duration of action effectively covers the critical early postoperative period, thereby promoting physical comfort and facilitating functional recovery (42). Effective pain control is widely regarded as a cornerstone of improved postoperative QoR (43). The use of LB not only provides sustained analgesia but also significantly reduces postoperative opioid consumption, thereby decreasing the incidence of common opioid-related adverse effects such as PONV, sedation, and gastrointestinal dysfunction (15, 44). These benefits contribute to enhanced emotional wellbeing and overall patient satisfaction, positively impacting multiple QoR domains. However, our analysis found no statistically significant differences in QoR scores at 24 and 48 h postoperatively. Several factors may explain this observation. First, despite the prolonged analgesic action of LB, its independent effect may be masked by the widespread use of multimodal analgesia in the immediate postoperative period. Second, early QoR scores may be influenced by transient perioperative stressors, including PONV, fatigue, and residual anesthetic effects, which could blunt the perceived benefit of LB. By 72 h, patients usually transition into a more stable recovery phase with fewer confounding factors, allowing the sustained analgesic effect of LB to manifest as a clinically meaningful improvement in QoR. Given the substantial heterogeneity observed in the pooled effects, subgroup analyses were performed to explore potential sources. The findings revealed that studies administering LB monotherapy yielded more consistent benefits than when combined with conventional local anesthetics. Geographical and administration route differences also contributed to variability—nerve blocks were more effective than infiltration, and studies from China showed the greatest effect sizes.

Importantly, subgroup analysis by surgical type showed that LB’s benefit was most pronounced in thoracic surgery, followed by abdominal surgery, with a minimal effect in orthopedic procedures. This highlights that LB’s efficacy may be context-dependent, influenced by pain mechanisms and baseline analgesic strategies. In addition, the route of administration appeared to be critical: nerve block administration significantly improved QoR at 72 h, whereas local infiltration showed no meaningful effect. These findings suggest that both the surgical context and the delivery method influence LB’s clinical effectiveness. However, the small number of studies in some subgroups limits definitive conclusions. Future studies should target specific surgical populations to clarify where LB offers the greatest clinical value.

Beyond surgical type, several clinical factors may account for the substantial heterogeneity observed in outcomes such as QoR at 72 h and postoperative pain scores. These include variations in anesthesia techniques, adherence to ERAS protocols, and perioperative care practices. Additionally, there were differences among control groups, including the use of placebo, standard bupivacaine or ropivacaine, with or without adjuncts (e.g., epinephrine, dexamethasone), and diverse administration methods. These factors likely contributed to variability in effect estimates and limited between-study comparability. Although subgroup analyses were performed where data allowed, incomplete reporting restricted further exploration. Future studies should aim for greater methodological consistency and standardized reporting to reduce heterogeneity and enhance evidence synthesis.

This study also observed that LB significantly reduced postoperative resting pain scores over three days, a result similar to that of Daher et al. (44). In a large-scale study involving 1,269 spinal surgery patients, Daher et al. (44) demonstrated that LB effectively reduced postoperative pain scores. In contrast, Hussain et al. (45), in their evaluation of LB for TAP block analgesia, found no superiority over conventional local anesthetics in terms of pain scores. The observed differences may be attributed to various factors, such as the type of surgery, LB dosage, regional anesthesia techniques, control group interventions, and postoperative pain management strategies. Almost all of the studies included by Hussain et al. (45) employed a multimodal analgesic approach, which may have masked the standalone benefits of LB. In contrast, several studies included in this analysis (31, 36, 37, 39) did not use multimodal analgesia. The combined analgesic effect of multimodal analgesia may have diminished the advantage of LB compared to the control group, which helps explain the positive outcomes observed in our analysis. LB, with its unique sustained-release delivery system, prolongs the duration of local anesthesia, offering effective support for early postoperative pain management (46).

Furthermore, although the overall meta-analysis did not show a statistically significant difference in cumulative morphine consumption over the first three postoperative days, sensitivity analysis suggested that a study using interscalene continuous nerve block as the control group might be the primary source of heterogeneity. The analgesic regimen used in the control group of that study provided strong pain relief, which may have masked the potential advantage of LB. After excluding this study, a re-analysis showed a significant reduction in cumulative morphine consumption in the LB group, and the heterogeneity dropped to 0%. However, these results should be interpreted with caution, as opioids are a major contributor to PONV, sedation, and delayed mobilization (47, 48). All of these factors impair recovery and lower QoR scores (49). PONV in particular affects multiple QoR domains, including physical comfort, appetite, and emotional wellbeing (50). Thus, alleviating PONV plays a crucial role in improving postoperative QoR (7). Additionally, we observed increased patient satisfaction in the LB group, further supporting its role in optimizing recovery. In conclusion, LB offers multiple clinical benefits by alleviating postoperative pain, reducing opioid-related adverse reactions, enhancing patient satisfaction, and improving overall recovery quality. It plays a crucial role in optimizing perioperative pain management strategies and enhancing the overall postoperative recovery experience.

Our study has several limitations: Firstly, we did not standardize the dosage of LB, local anesthesia techniques, or the interventions used in the control groups. Additionally, the volume of LB solution varied across the included studies, which may have contributed to the higher heterogeneity and influenced the analgesic effects. Secondly, we did not assess factors such as postoperative infections, cardiopulmonary complications, or hospital stay, as these data were unavailable or not reported in the included studies. This may limit the comprehensiveness of our findings, as such factors could independently affect QoR. Thirdly, QoR scores are self-reported, which may introduce subjectivity and potential measurement error. The potential for sponsorship bias should also be considered in studies involving proprietary LB formulations.

## 5 Conclusion

This meta-analysis suggests that LB may improve postoperative recovery quality within 72 h (based on moderate-quality evidence), likely through mechanisms such as reducing postoperative morphine use (moderate-quality evidence), lowering the incidence of PONV (high-quality evidence), and enhancing patient satisfaction (low quality evidence). However, due to the heterogeneity among the studies and the limited sample sizes for some outcomes, these findings should be interpreted with caution. Further high-quality, large-sample RCTs are needed to confirm these results, particularly for outcomes with low or moderate certainty.

## Data Availability

The original contributions presented in this study are included in this article/Supplementary material, further inquiries can be directed to the corresponding author.
