# Supplementary material for: Impact of liposomal bupivacaine on subjective recovery quality after surgery: a meta-analysis of randomized controlled trials
Source: Front Med (Lausanne). 2025 Oct 8;12:1655756. doi: 10.3389/fmed.2025.1655756 (PMC12540450; doi:10.3389/fmed.2025.1655756)
Supplement: Supplementary file 1 [file Data_Sheet_1.docx]

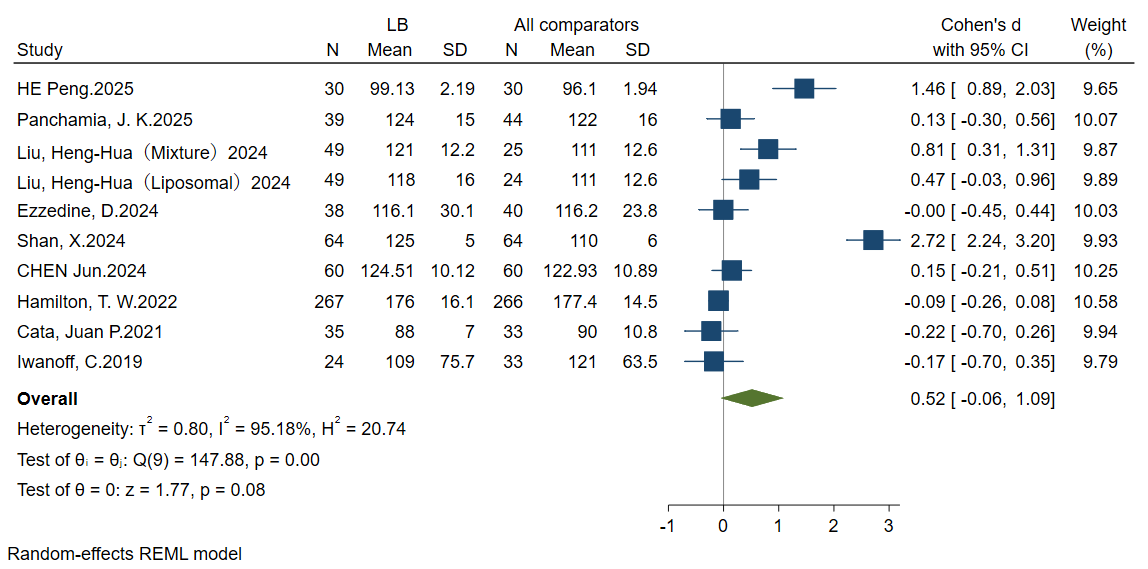


**Supplementary figure 1** Forest plot showing QoR within 24 hours after surgery，SD: standard deviation CI: confidence interval.


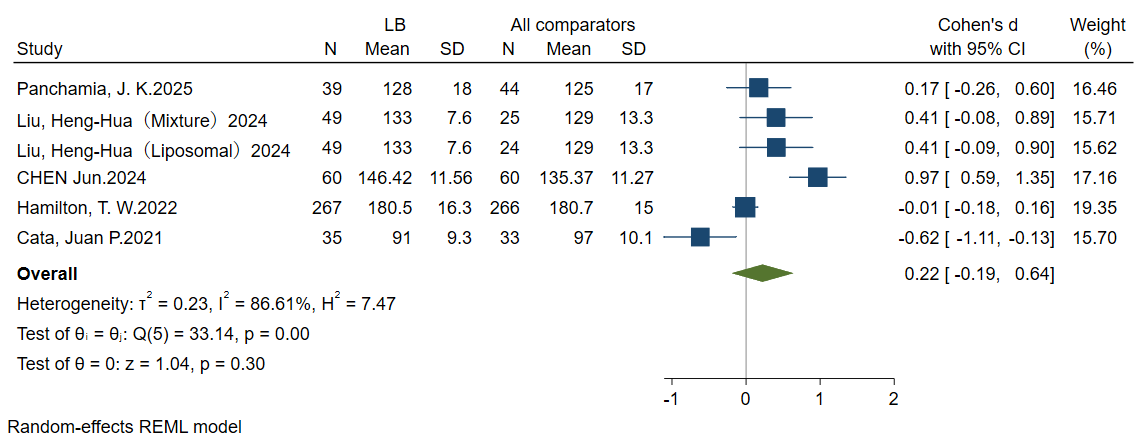


**Supplementary figure 2** Forest plot showing QoR within 48 hours after surgery，SD: standard deviation CI: confidence interval.


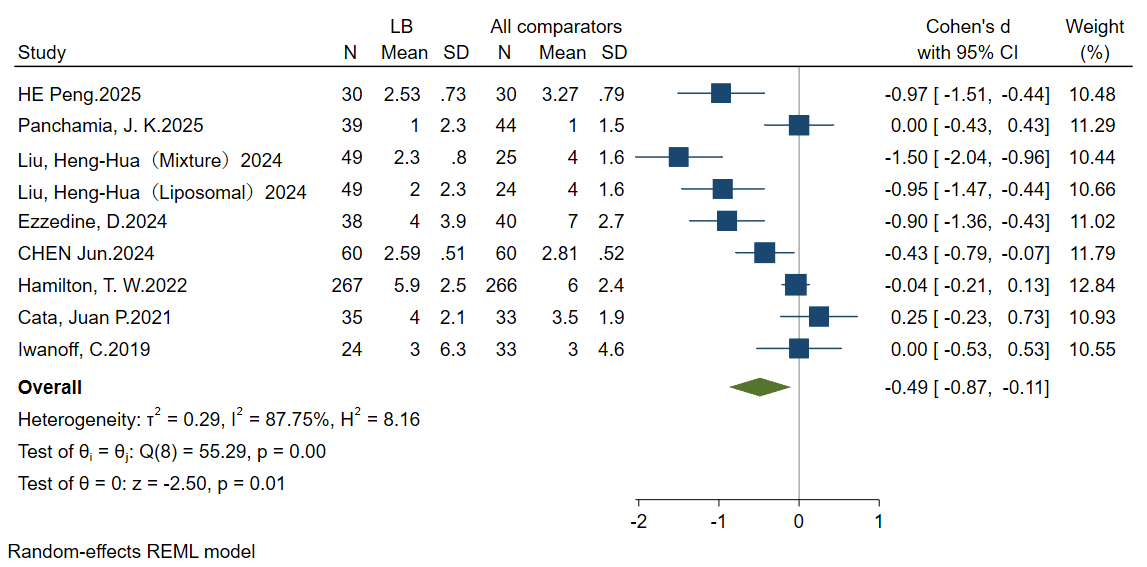


**Supplementary figure 3** Forest plot showing resting pain scores at 24 hours after surgery. SD: standard deviation; CI: confidence interval.


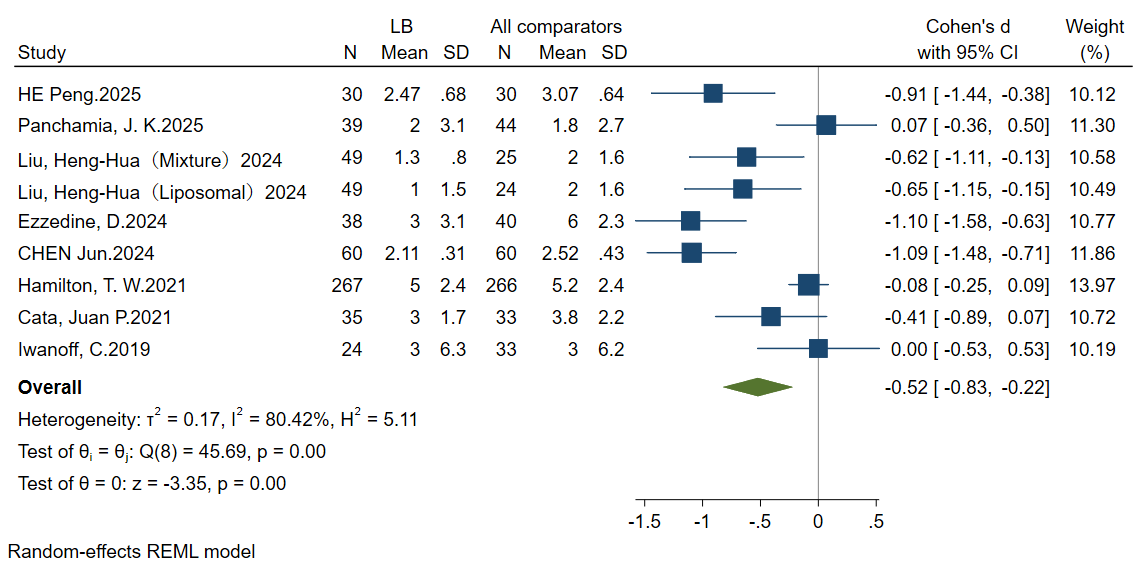


**Supplementary figure 4** Forest plot showing resting pain scores at 48 hours after surgery. SD: standard deviation; CI: confidence interval.


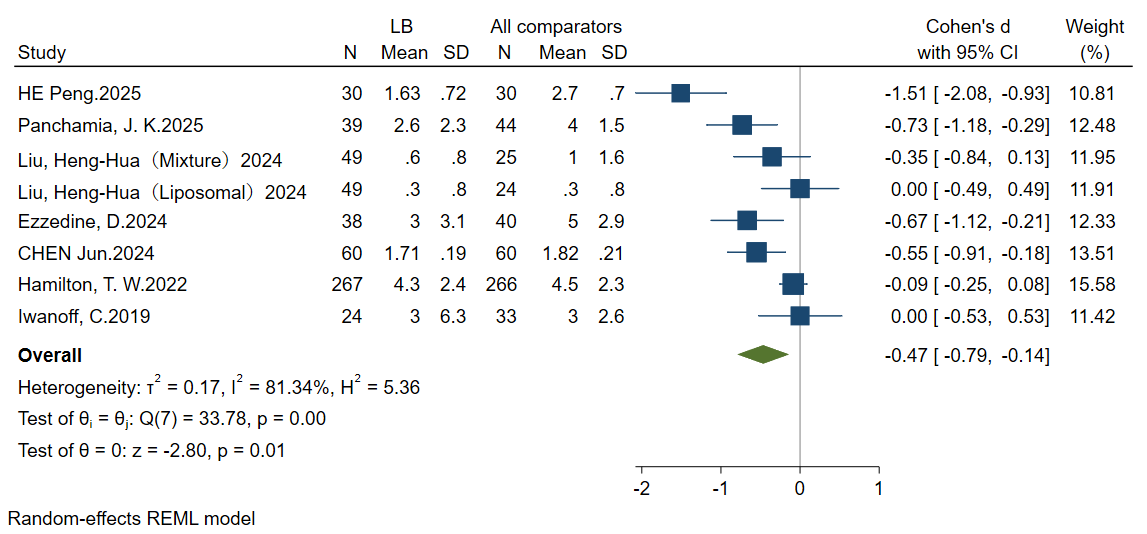


**Supplementary figure 5** Forest plot showing resting pain scores at 72 hours after surgery. SD: standard deviation; CI: confidence interval.


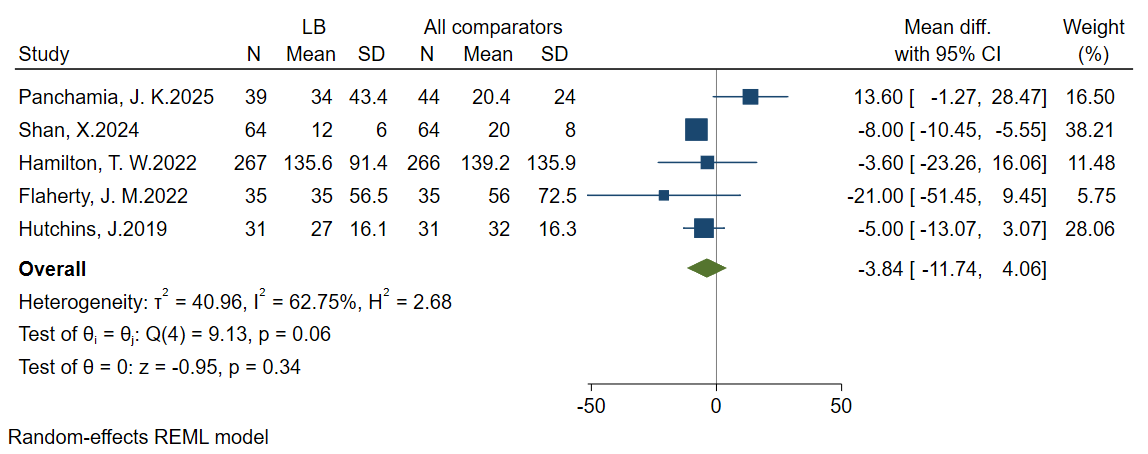


**Supplementary figure 6** Forest plot showing cumulative morphine consumption over three days postoperatively. SD: standard deviation; CI: confidence interval.


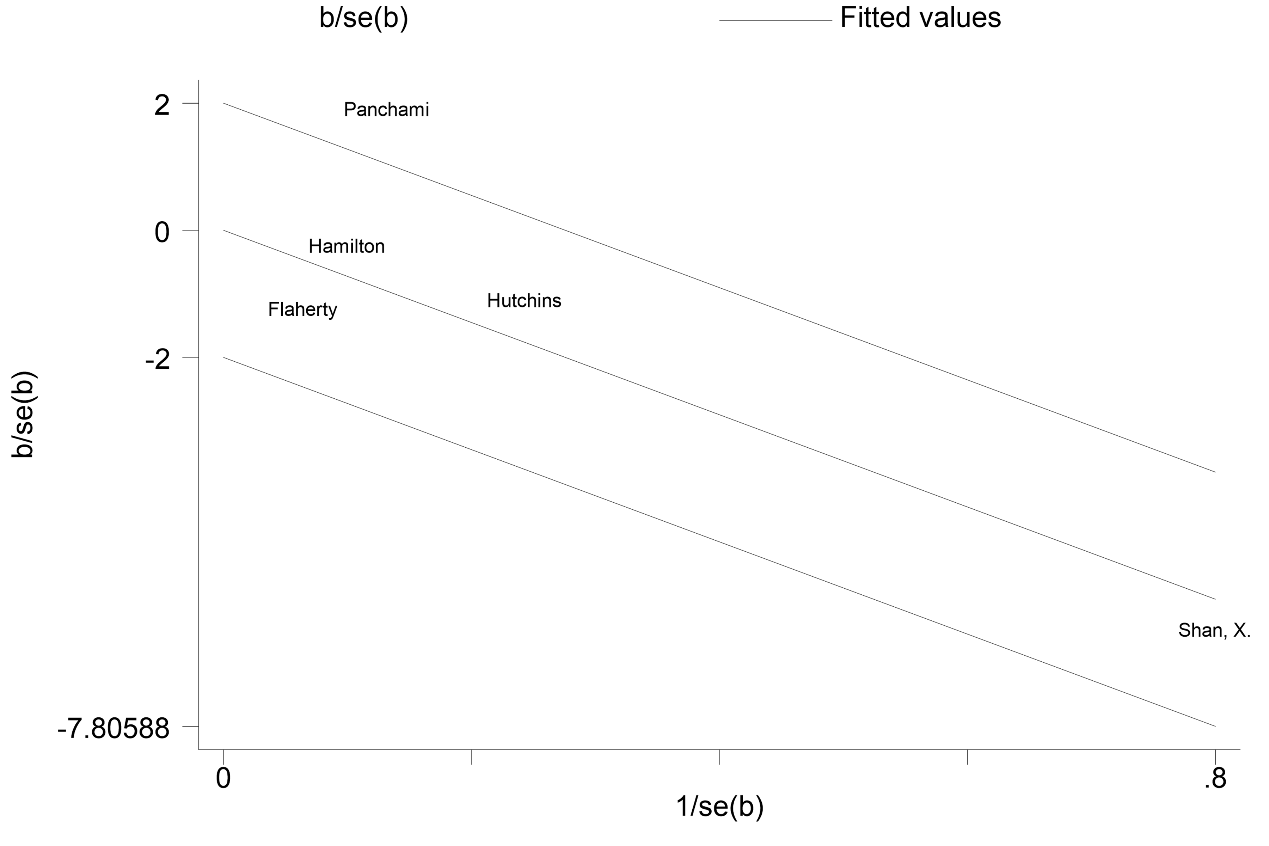


**Supplementary figure 7** Galbraith plot of cumulative morphine consumption over three days postoperatively


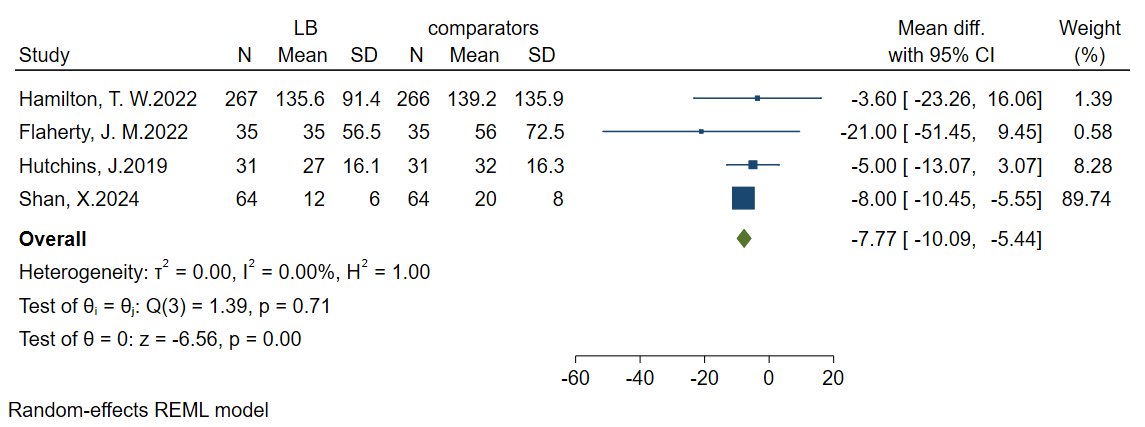


**Supplementary figure 8** Forest plot showing cumulative morphine consumption over three days postoperatively. SD: standard deviation; CI: confidence interval.


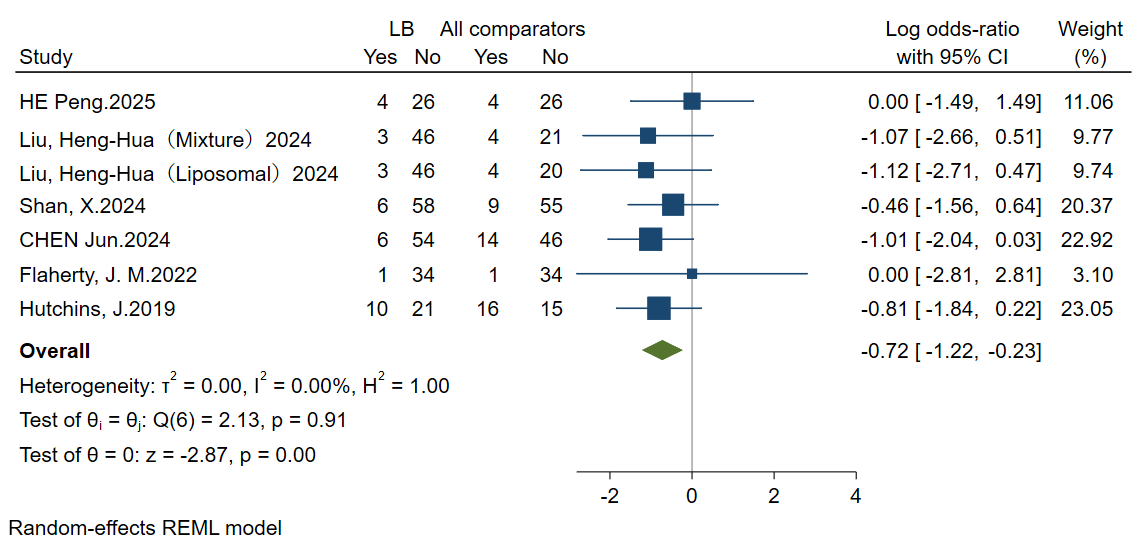


**Supplementary figure 9** Forest plot showing PONV incidence. SD: standard deviation; CI: confidence interval.


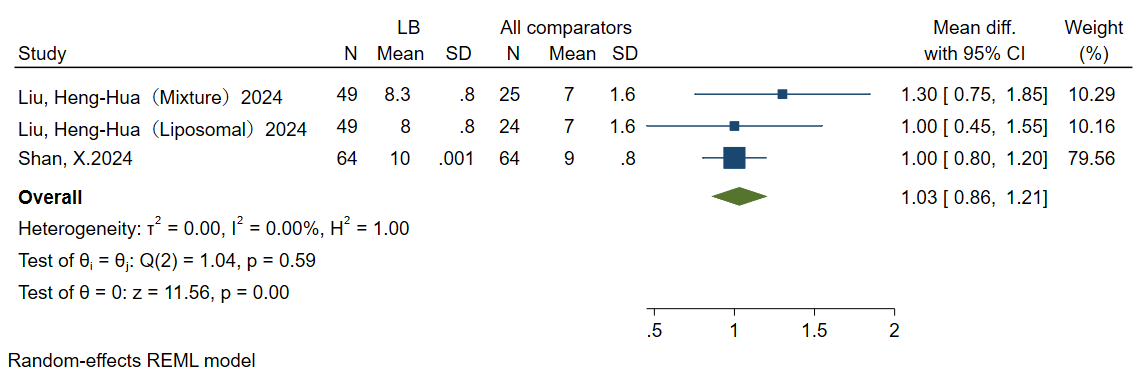


**Supplementary figure 10** Forest plot showing patient satisfaction; CI: confidence interval.


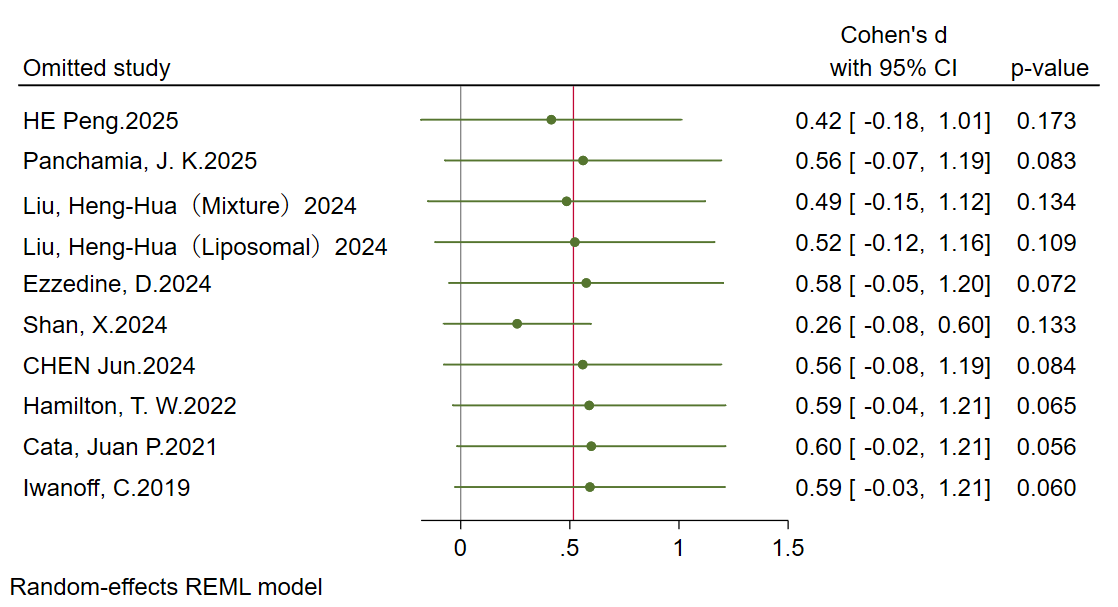


**Supplementary figure 11** leave-one-out approach for postoperative 24-hour recovery quality


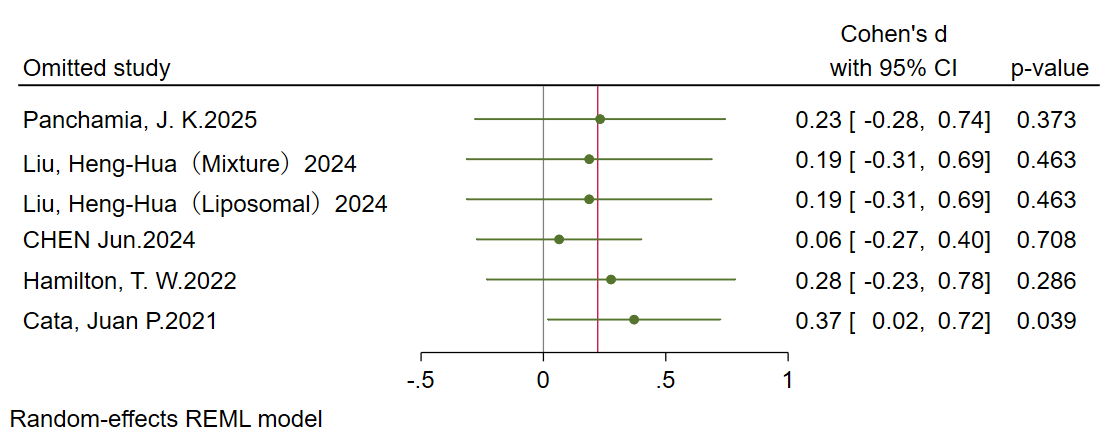


**Supplementary figure 12** leave-one-out approach for postoperative 48-hour recovery quality


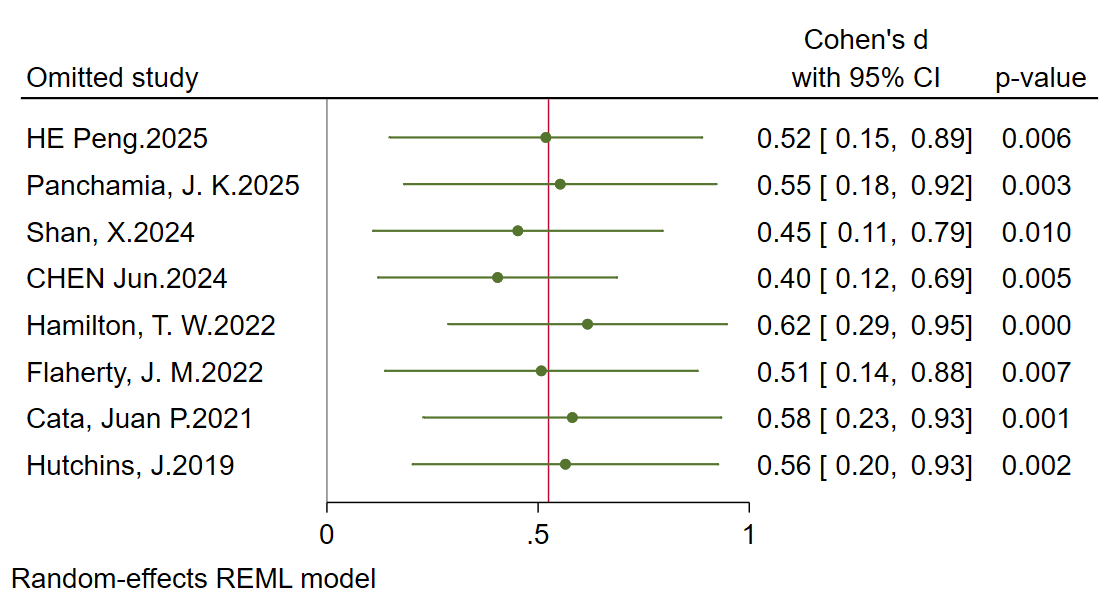


**Supplementary figure 13** leave-one-out approach for postoperative 72-hour recovery quality


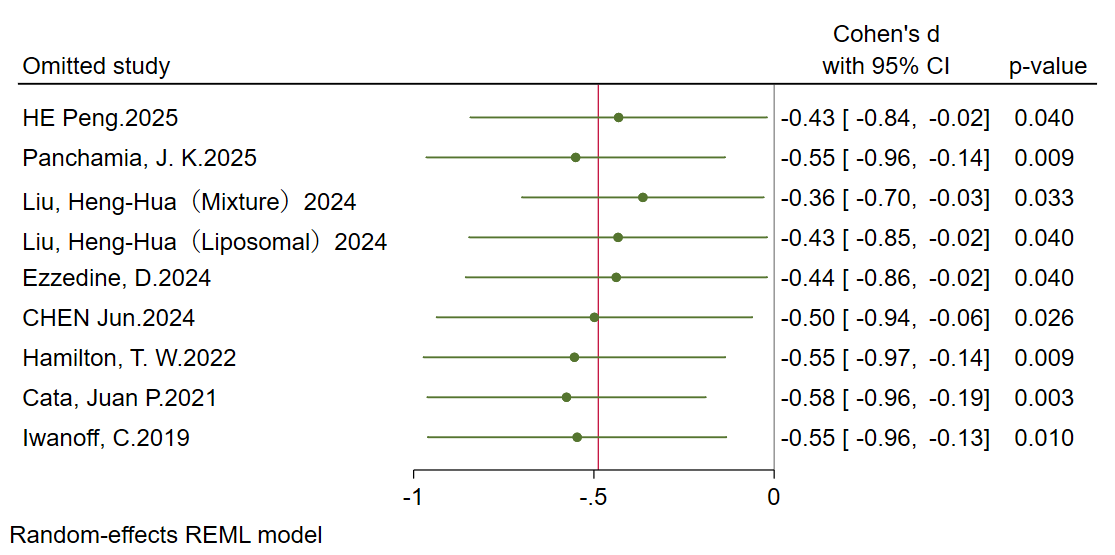


**Supplementary figure 14** leave-one-out approach for resting pain scores at 24 hours postoperatively.


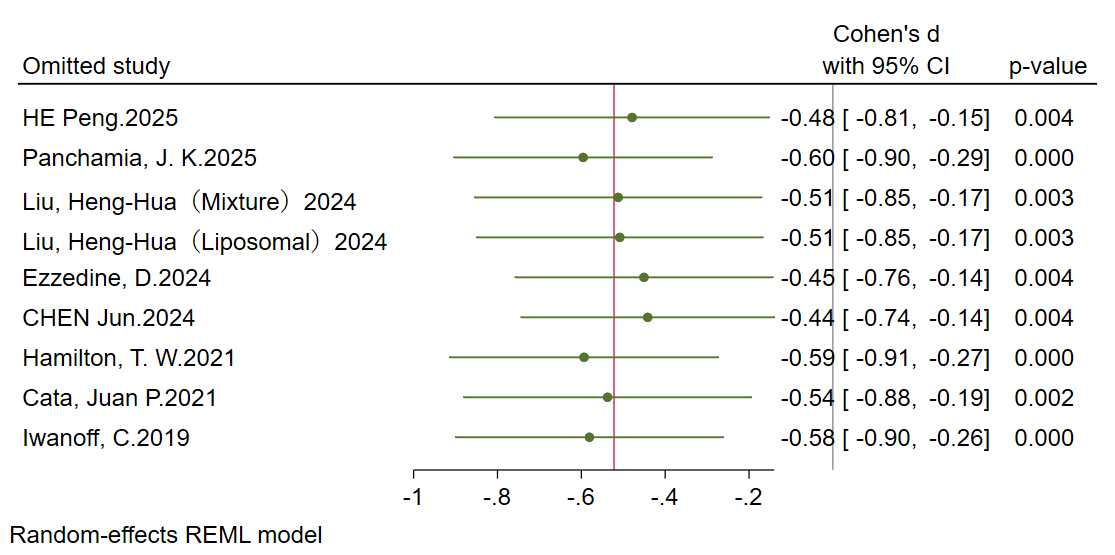


**Supplementary figure 15** leave-one-out approach for resting pain scores at 48 hours postoperatively.


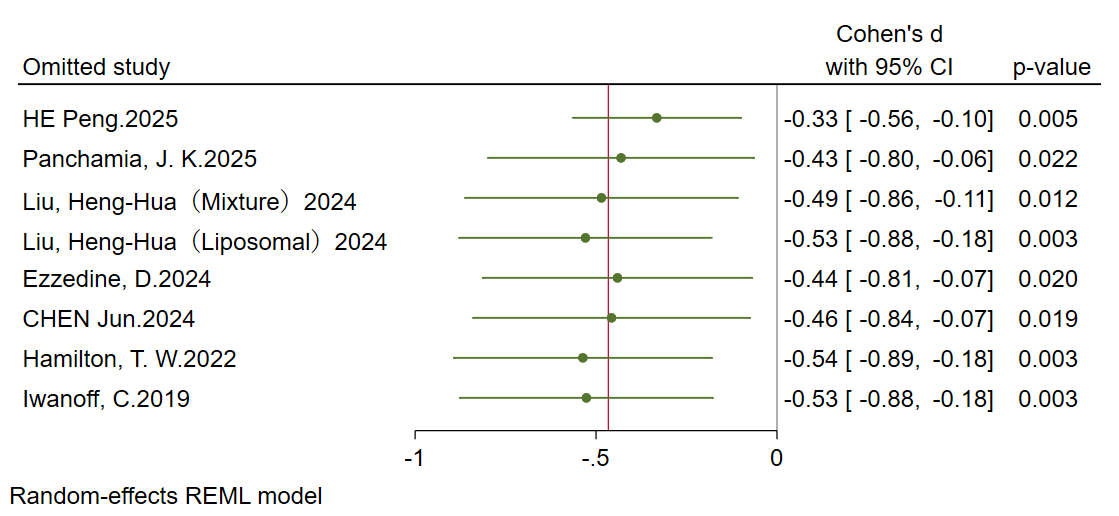


**Supplementary figure 16** leave-one-out approach for resting pain scores at 72 hours postoperatively.


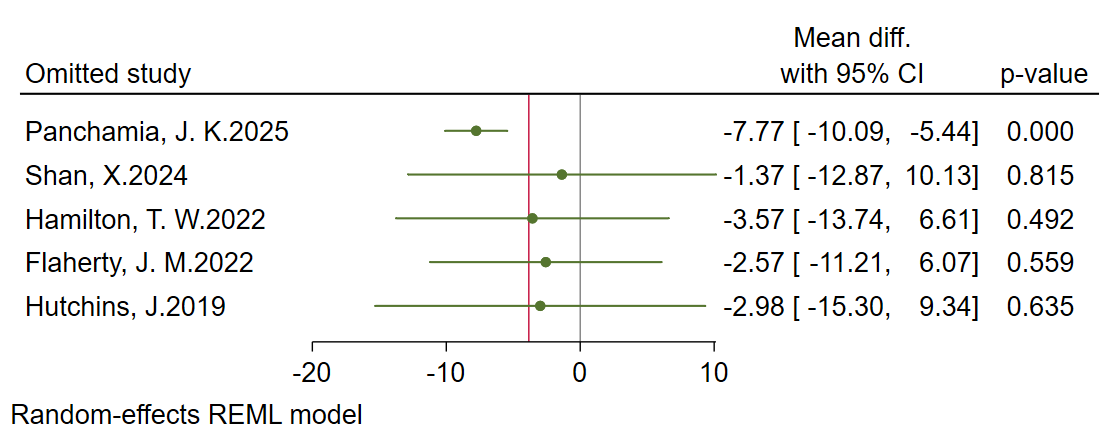


**Supplementary figure 17** leave-one-out approach for cumulative morphine consumption over three days postoperatively.


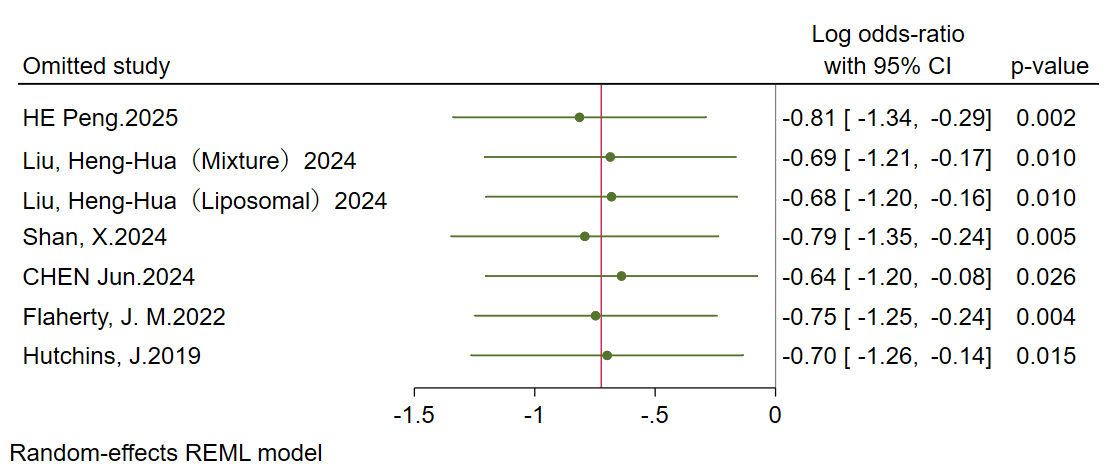


Supplementary figure 18 leave-one-out approach for PONV incidence


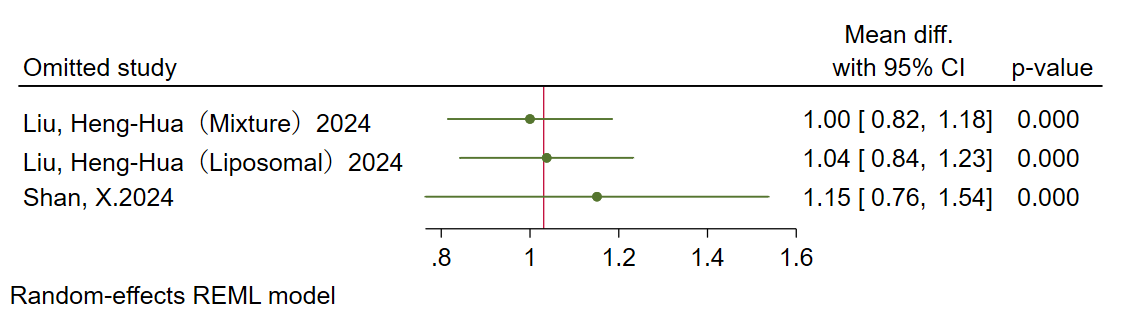


**Supplementary figure 19** leave-one-out approach for patient satisfaction
